# Coronary Artery Disease Presentation and Its Association with Shortened Activated Partial Thromboplastin Time

**Published:** 2018-01

**Authors:** Maryam Sotoudeh Anvari, Mojgan Tavakoli, Masoumeh Lotfi-Tokaldany, Mohammadali Broumand, Omid Rezahosseini, Elham Hakki-Kazzazi, Arash Jalali

**Affiliations:** *Tehran Heart Center, Tehran University of Medical Sciences, Tehran, Iran. *

**Keywords:** *Blood coagulation tests*, *Partial thromboplastin time*, *Prothrombin time*, *Coronary artery disease*, *Myocardial infarction*

## Abstract

**Background: **Standard coagulation screening tests are important constituents of basic examinations in clinical laboratories. There is no clear evidence of a relation between the type of clinical presentation and coagulation parameters in patients with suspected coronary artery disease.

**Methods: **This cross-sectional study included 539 patients who underwent coronary angiography in Tehran Heart Center between November 2012 and January 2013. Patients presented with ST-segment-elevation myocardial infarction (STEMI), non-STEMI, unstable angina, or stable angina. Prothrombin time (PT), international normalized ratio (INR), and activated partial thromboplastin time (APTT) were measured before angiography and compared between the clinical presentation groups.

**Results: **The mean age of the patients was 59.156 ± 11.05 years, and 47.7% were male. STEMI was reported in 41(7.6%) patients, non-STEMI in 42 (7.8%), unstable angina in 304 (56.4%), and stable angina in 152 (28.2%). No difference in the mean PT and INR was found between the groups. The mean APTT was significantly lower among the patients presenting with STEMI and non-STEMI (26.58 ± 2.32 s in the STEMI, 26.85 ± 2.41 s in the non-STEMI, 27.64 ± 2.54 s in the unstable, and 27.93 ± 2.53 s in the stable angina groups, respectively, p value = 0.005). After adjustment, the association between the patients’ presentations and APTT was significant (OR for 5 s increase in APTT = 1.661, 95% CI = 1.184 to 2.332; p value = 0.003).

**Conclusion: **We observed that the patients who presented with STEMI had the lowest value of APTT, whereas those who presented with stable angina had the highest. The value of APTT in patients undergoing coronary angiography may have a potential to predict the extent and severity of coronary stenosis.

## Introduction

Standard coagulation screening tests such as activated partial thromboplastin time (APTT), prothrombin time (PT), and the international normalized ratio (INR) are important constituents of basic examinations in clinical laboratories. APTT can be used as an indicator of intrinsic coagulation pathway activity,^   1 ^ and a short APTT is linked to increased thrombin generation and increased risk for thrombosis.^   2 ^

Previous studies have demonstrated the role of blood clotting activation such as elevated circulating levels of a marker of thrombin generation and fibrinopeptide A in the occurrence of angina in ischemic heart diseases.^3^^-^^5^ An association has also been reported between environmental factors, hemostatic alterations, inflammation, and vascular events.   6  In addition, common hemostatic markers have been found to be correlated with an increase in the generation of thrombin and the development of prothrombotic state.^   2 ^

The spectrum of coronary artery disease (CAD) presentation ranges from the stable condition, known as “stable angina pectoris”, to the acute form-comprising unstable angina, non–ST-elevation myocardial infarction (NSTEMI), and ST-elevation myocardial infarction (STEMI).^7^^-^^9^ It is not clear whether the blood level of hemostatic markers can be used for the screening of individuals who are at risk for acute coronary syndrome. 

We sought to assess the relation between the type of clinical presentation and coagulation parameters in patients with suspected CAD who underwent coronary artery angiography with a view to introducing a commonly usable and inexpensive indicator for the prediction of CAD manifestations.

## Methods

This cross-sectional study recruited all patients referred to the Angiography Department of Tehran Heart Center for CAD workup between November 7, 2012, and January 7, 2013. All the patients’ demographic data and past medical history were recorded, and their PT, INR, and APTT were measured before angiography. Eligible patients were those whose indication for angiography was typical chest pain. Patients with a history of cardiac surgery or coronary artery stenting, epicardial coronary artery narrowing less than 50% in the absence of significant coronary artery stenosis, coagulopathic disorders, renal failure, and use of anticoagulants (warfarin and heparin) in the preceding month were excluded. Finally, 539 patients were incorporated in the final analysis. The study design was approved by the Scientific Committee of Tehran Heart Center, and signed informed consent was obtained from all the patients allowing the hospital’s researchers to use their medical information for research purposes.

For PT and APTT, blood was collected in the standard condition (i.e., in 3.2% sodium citrate [light blue cap] tubes with a blood-to-anticoagulant ratio of 9:1). Afterward, plasma was prepared by centrifugation for 10 minutes at 1500 rpm at room temperature. PT and APTT were measured using an automated optical coagulometer (ELITE system [Instrumentation Laboratory, Italy]) and a SynthASil reagent kit for APTT (RecombiPlasTin 2G reagent kit), with an international sensitivity index less than 1 for PT from the same company. All the measurements were made using a single lot number of the reagent. The results are shown as seconds for PT and APTT.

Internal quality control of the results was done twice a day by grading them as normal, low abnormal, and high abnormal and external quality control was done by comparing the results with those of peer groups in a proficiency-testing program. 

The definitions of hyperlipidemia, diabetes mellitus, and smoking were adopted according to Tehran Heart Center’s Angiography Database which were published previously.^10^

The presenting symptoms were categorized at 4 levels: stable angina, unstable angina, NSTEMI, and STEMI. Severity of presentation increasing from stable angina toward STEMI was considered the ordinal outcome. CAD was defined as luminal narrowing equal to or greater than 50% in at least 1 epicardial coronary artery. 

The continuous variables are presented as means ± standard deviations (SDs), and they were compared between the CAD groups using the Student* t*-test or the Mann-Whitney U test. Also, they were compared between the levels of the mentioned ordinal outcomes via the Kruskal-Wallis test. The categorical variables are expressed through frequencies and percentages, and they were compared between the levels of the ordinal outcomes using the χ^2^ test or the Fisher exact test. The associations between PT, APTT, and the INR and the clinical presentation groups were adjusted in terms of age, gender, body mass index, diabetes mellitus, cigarette smoking, and blood creatinine level-which were detected as possible confounders-using multiple logistic regression models. The relations between PT, APTT, and the INR and the ordinal outcomes were adjusted as regards the potential confounders using cumulative logit models. A p value equal to or less than 0.05 was considered statistically significant. The statistical analyses were done using IBM SPSS Statistics for Windows, version 20.0 (Armonk, NY: IBM Corp.). 

## Results

In our study population of 539 patients (47.7% male, mean age = 59.156 ± 11.05 y), the indications for angiography were STEMI in 41 (7.6%) patients, NSTEMI in 42 (7.8%), unstable angina in 304 (56.4%), and stable angina in 152 (28.2%). Out of the 539 patients, 301 (55.8%) had CAD. The baseline continuous and categorical characteristics of the patients, according to their clinical presentation, are listed in Table 1. 

**Figure 1:**
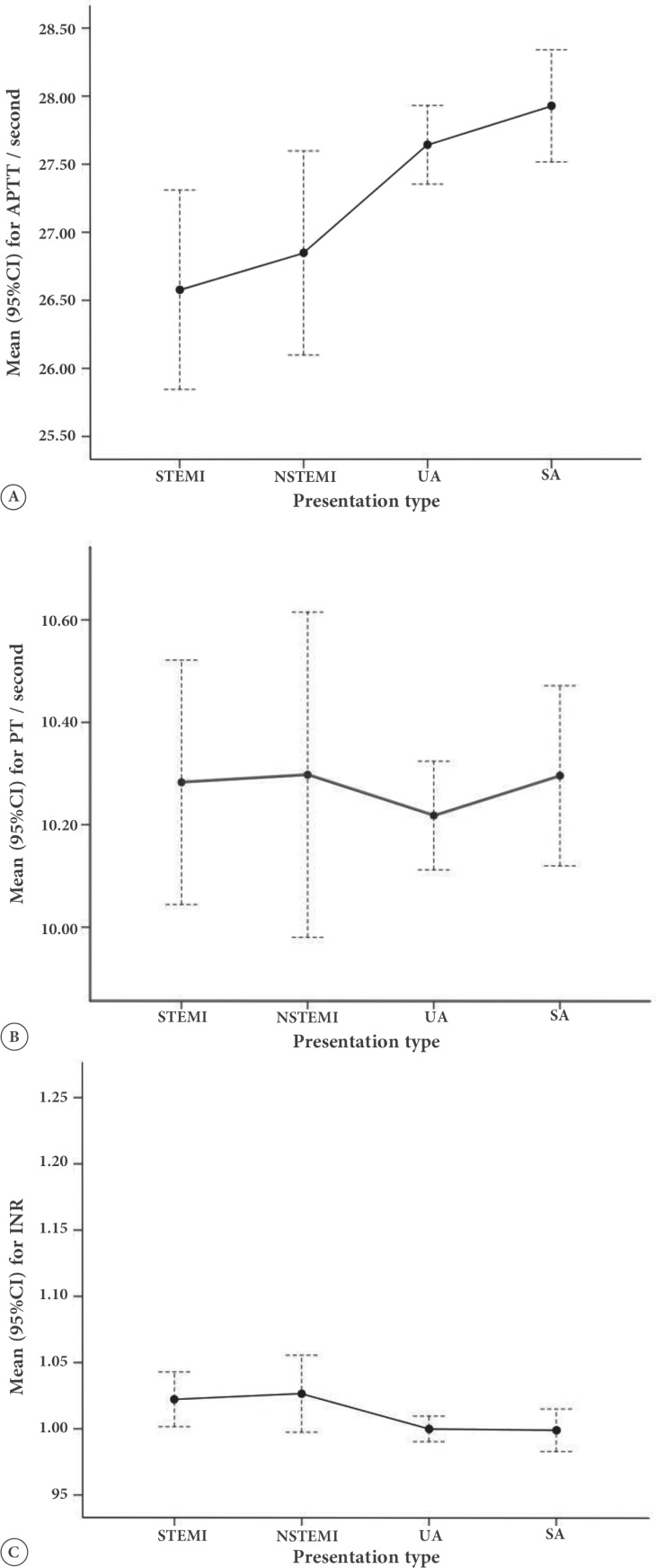
Comparison of the levels of different coagulation parameters across different types of clinical presentations in the patients undergoing coronary angiography.

PT, Prothrombin time; APTT, Activated partial thromboplastin time; INR, International normalized ratio, STEMI, ST-segment elevation myocardial infarction; NSTEMI, Non–ST-segment elevation myocardial infarction; UA, Unstable angina; SA, Stable angina

The patients with STEMI had the lowest frequency of the female gender (19.5%), while the stable angina group had the highest proportion of women (59.9%). The mean of age decreased significantly as we moved from extremely acute STEMI toward the most stable condition (i.e., stable angina). The frequency of cigarette smoking, either in the form of current smoker or former smoker, decreased significantly from the STEMI group toward the stable angina group. Diabetes mellitus was significantly more frequent among the patients presenting with NSTEMI. Based on coronary angiography, the diagnosis of coronary artery stenosis was established more commonly in the patients with STEMI or NSTEMI and less frequently in those with unstable angina or stable angina. Regarding the coagulation parameters, no difference in the mean PT and INR was found between the groups, while the mean APTT was significantly lower among the patients presenting with STEMI or NSTEMI than among those presenting with unstable angina or stable angina (Figure 1).

According to the ordinal logistic regression analysis after adjustment for the potential confounders (Table 2), the relations between the type of clinical presentation and APTT were statistically significant (OR for a 5-s increase in APTT = 1.661, 95%CI: 1.184 - 2.332; p value = 0.003). The odds of a 1-grade increase in the severity of presentation became 1.66 times higher with a 5-s increase in APTT. Moreover, PT and the INR were not correlated with the type of presentation (Table 2).

**Table 1:** Baseline demographic and clinical characteristics of the patients*

	STEMI (n=41)	NSTEMI (n=42)	UA (n=304)	SA (n=152)	P value
Demographics					
Female gender	8 (19.5)	19 (45.2)	164 (53.9)	91 (59.9)	< 0.001
Age (y)	60.93±8.79	64.24±12.40	59.41±10.73	56.78±11.32	0.001
BMI (kg/m^2^)	28.20±4.45	27.76±4.37	28.96±4.77	29.81±5.49	0.054
Abdominal waist (cm)	100.05±7.22	100.33±12.22	102.71±9.82	104.39±11.93	0.043
Risk Factors					
Positive history of MI	11 (26.8)	9 (21.4)	52 (17.1)	25 (16.4)	0.405
Positive familial history	8 (19.5)	8 (19.0)	59 (19.5)	28 (18.7)	0.997
Smoking					0.005
Current	8 (19.5)	7 (16.7)	44 (14.5)	14 (9.2)	
Former	11 (26.8)	7 (16.7)	52 (17.1)	12 (7.9)	
Hyperlipidemia	31 (75.6)	36 (85.7)	246 (80.9)	116 (76.3)	0.439
Hypertension	27 (65.9)	30 (71.4)	202 (66.4)	91 (59.9)	0.422
Diabetes mellitus	13 (31.7)	23 (54.8)	102 (33.6)	47 (30.9)	0.033
CAD	37 (90.2)	35 (83.3)	171 (56.3)	58 (38.2)	< 0.001
Lab Findings					
Creatinine (mg/dL)	1.07±0.37	1.08±0.35	0.90±0.27	0.86±0.28	< 0.001
PT (sec)	10.28±0.76	10.30±1.02	10.22±0.94	10.30±1.10	0.850
INR	1.02±0.07	1.03±0.09	1.00±0.09	1.00±0.10	0.140
APTT (sec)	26.58±2.32	26.85±2.41	27.64±2.54	27.93±2.53	0.005

STEMI, ST-segment elevation myocardial infarction; NSTEMI, Non-ST-segment elevation myocardial infarction; UA, Unstable angina; SA, Stable angina; BMI, Body mass index; MI, Myocardial infarction; CAD, Coronary artery disease; PT, Prothrombin time; INR, International normalized ratio; APTT, Partial thromboplastin time

* Data are presented as mean±SD or n (%).

**Table 2:** Adjusted associations between 3 coagulation factors and the type of clinical presentation

	OR	95% CI	P value
APTT (for 5-s increase)	1.661	1.184 - 2.332	0.003
PT (for 5-s increase)	1.016	0.427 - 2.415	0.971
PTT (for 5-s increase)	0.326	0.048 - 2.207	0.251

APTT, Activated partial thromboplastin time; PT, Prothrombin time; INR, International normalized ratio

## Discussion

The results of the present study revealed that among 3 coagulation factors (PT, APTT, and the INR), APTT was correlated significantly with the type of clinical presentation in the patients candidated for coronary angiography. The patients presenting with STEMI or NSTEMI had a significantly lower mean of APTT. We found no relationship between the measured PT, APTT, and the INR. 

The pathogenesis of local thrombus formation on a ruptured plaque and hypercoagulable state during an acute coronary event has been described before.^11^ Variables such as local rheological conditions, systemic thrombogenic factors, diabetes, and hypercoagulable state may influence the degree and duration of thrombus formation.^   11^

Previous studies have demonstrated that an abnormally shortened APTT is associated with an increased risk for venous thromboembolism.^12^^-^^14^ However; limited studies have focused on the relation between a shortened APTT and arterial thrombosis. Abdullah et al.^15^ reported that a shortened APTT was correlated with acute arterial thrombosis in patients presenting with acute coronary syndrome and suggested that the presence or absence of an abnormally short APTT in patients with suspected acute coronary syndrome could be used as a negative or positive predictive marker to support the clinical diagnosis. Our findings chime in with the results of the study by Abdullah and colleagues.^        15 ^ In our study, the mean of APTT was significantly lower in the patients with STEMI or NSTEMI than in those with unstable angina or stable angina, showing that the severity of patient presentation was correlated with a shorter APTT. Nevertheless, such association could not be detected with respect to the value of PT and the INR. This may be related to the process of thrombosis formation, which is allied with the internal hemostatic pathway.

Martin et al.^16^ retrospectively studied 592 patients who were admitted to the emergency department with chest pain and had coagulation samples. Reporting that only 79 patients had abnormal coagulation tests-predictable on the basis of a history of warfarin or heparin use or a history of liver disease-the authors concluded that routine coagulation testing in adults presenting to the emergency department with chest pain was unnecessary. In contrast to their study, in the present study we excluded all the patients with a history of cardiac surgery or coronary artery stenting, coagulopathic disorders, renal failure, and use of anticoagulants (warfarin and heparin) and observed that in our selected group of patients, APTT was correlated significantly with STEMI and NSTEMI. 

Our data also confirm the findings by Lytvyn et al.,^17^ who examined the correlation between inflammatory factors (IL-1beta and IL-6) and thrombosis (von Willebrand factor) and suggested a positive correlation between inflammation and the thrombosis markers measured on admission in patients with either Q-wave or non–Q-wave myocardial infarction. 

First and foremost among the limitations of the present study is that our conclusions could have become more robust had we been able to measure additional coagulation markers such as Factor VIII. 

## Conclusion

Our results revealed that APTT was significantly correlated with the type of clinical presentation in patients candidated for coronary angiography. Moreover, the patients presenting with STEMI or NSTEMI had a significantly lower mean of APTT. APTT may be a useful marker to screen patients prone to present with acute coronary syndrome and may also help manage acute coronary syndrome. Future studies are needed to determine the clinical importance of a reduced APTT in patients undergoing coronary angiography. 

## Notes:

***This paper should be cited as:*** Sotoudeh Anvari M, Tavakoli M, Lotfi-Tokaldany M, Broumand M, Rezahosseini O, Hakki-Kazzazi E, Jalali A. Coronary Artery Disease Presentation and Its Association with Shortened Activated Partial Thromboplastin Time. J Teh Univ Heart Ctr 2018;13(1):1-5.
